# Zebrafish Circadian Clock Entrainment and the Importance of Broad Spectral Light Sensitivity

**DOI:** 10.3389/fphys.2020.01002

**Published:** 2020-08-14

**Authors:** Inga A. Frøland Steindal, David Whitmore

**Affiliations:** ^1^Centre for Cell and Molecular Dynamics, Department of Cell and Developmental Biology, University College London, London, United Kingdom; ^2^College of Public Health, Medical and Veterinary Sciences, Department of Molecular and Cell Biology, James Cook University, Townsville, QLD, Australia

**Keywords:** zebrafish, entrainment, opsin, non-visual photopigment, circadian clock, phase shift, monochromatic light

## Abstract

One of the key defining features of an endogenous circadian clock is that it can be entrained or set to local time. Though a number of cues can perform this role, light is the predominant environmental signal that acts to entrain circadian pacemakers in most species. For the past 20 years, a great deal of work has been performed on the light input pathway in mammals and the role of intrinsically photosensitive retinal ganglion cells (ipRGCs)/melanopsin in detecting and sending light information to the suprachiasmatic nucleus (SCN). In teleost fishes, reptiles and birds, the biology of light sensitivity is more complicated as cells and tissues can be directly light responsive. Non-visual light signalling was described many years ago in the context of seasonal, photoperiodic responses in birds and lizards. In the case of teleosts, in particular the zebrafish model system, not only do peripheral tissues have a circadian pacemaker, but possess clear, direct light sensitivity. A surprisingly wide number of opsin photopigments have been described within these tissues, which may underpin this fundamental ability to respond to light, though no specific functional link for any given opsin yet exists. In this study, we show that zebrafish cells show wide spectral sensitivities, as well as express a number of opsin photopigments – several of which are under direct clock control. Furthermore, we also show that light outside the visual range, both ultraviolet and infrared light, can induce clock genes in zebrafish cells. These same wavelengths can phase shift the clock, except infrared light, which generates no shift even though genes such as *per2* and *cry1a* are induced.

## Introduction

The most ancient and predictable environmental cue for life on Earth is the onset of sunrise and sunset. In fact, it is hard to imagine any other environmental stimulus that an animal or plant experiences that lacks any biologically significant noise. As a consequence, most life under the Sun, from bacteria to plants to humans have evolved a circadian clock which internally represents this highly predictable change in day and night. A critical aspect of having such a circa 24-hour pacemaker is that it needs to be entrained or set each day to the environmental light-dark cycle. Natural selection acts on correct phase relationships rather than clock period, such that it is essential both internal and external oscillations are appropriately phase aligned. Consequently, entrainment is an essential and defining feature of the circadian clock and the topic addressed in this study.

Light sensing and entrainment were always thought to be a process associated exclusively with the eyes and the Suprachiasmatic Nucleus (SCN) in mammals, and the pineal gland in non-mammalian vertebrates (Suburo and de Iraldi, 1969; Ibuka and Kawamura, 1975; Elliott, 1976; Underwood and Groos, 1982; Cahill, 1996). It therefore came as a surprise, some 20 years ago, that the process of non-visual photoreception is something that all tissues in the zebrafish are capable of Whitmore et al. (1998). Although the zebrafish pineal has key functions (Ben-Moshe et al., 2014; Livne et al., 2016), teleost clock systems appear to be highly decentralised, with all tissues and the majority of cells possessing a directly light entrainable circadian pacemaker (Whitmore et al., 1998, 2000; Carr and Whitmore, 2005; Tamai et al., 2005; Steindal and Whitmore, 2019).

Peripheral light sensitivity is not exclusive to zebrafish, as most non-mammalian vertebrates such as fish, reptiles and birds show high opsin diversity (Davies et al., 2015). Deep-brain photoreception has been researched in avian seasonal physiology for many years, as has similar hypothalamic responses in reptiles (Benoit, 1935a, b; Underwood and Menaker, 1976; Takahashi and Menaker, 1979; Underwood and Groos, 1982; Wyse and Hazlerigg, 2009). So, perhaps it should not have come as such a surprise when this well-established direct brain light-sensitivity was expanded to include the majority of other tissues. Monotremes and mammals also express non-visual opsins that facilitate a range of biological processes, of which, melanopsin in mammalian clock entrainment is the most explored (Provencio et al., 1998; Halford et al., 2001; Tarttelin et al., 2003).

When peripheral photoreception was discovered in anamniotes, the next obvious question concerns the nature of the photopigment responsible for this peripheral light detection and clock entrainment? Visual photopigments have been studied extensively since the 19th century (Norris, 1895; Arey, 1915). However, a whole century past before science turned its interest to the discovery of the non-visual photopigments, and several candidates appeared through the late 1990s and early 2000s (Okano et al., 1994; Blackshaw and Snyder, 1997; Soni and Foster, 1997; Sun et al., 1997; Provencio et al., 1998). The number of opsins discovered since the early 1990s has increased to include 32 non-visual and 10 visual opsins in zebrafish (Davies et al., 2015), and new opsins, splice variants and isoforms are discovered in new species on a regular basis (Musilova et al., 2019). The non-visual and visual opsins are divided into 8 classes based on photoisomerase activity, molecular function and how they couple and signal though G-proteins. What sets several of the non-visual opsins apart from the visual opsins, is that they are bistable, meaning that instead of bleaching like the visual opsins, the photoproduct can convert between photoproduct and photopigment without releasing the chromophore (Tsukamoto, 2014). Such a process makes sense in the context of a tissue that lacks the more sophisticated pigment-regeneration mechanisms found in the retina.

With such a large diversity of opsins, identifying key candidates in the fish for photoentrainment of the clock, or how these photopigments work synergistically together is now more complicated than ever. Absorption spectra has been performed on many of these zebrafish opsins. Most are monophasic, but seemingly with somewhat broad absorption peaks, with most opsins absorbing in the blue-green, while some absorb up in the red end of the spectrum (Su et al., 2006; Davies et al., 2011, 2015; Koyanagi et al., 2015; Morrow et al., 2016; Sato et al., 2016, 2018; Sugihara et al., 2016; Steindal and Whitmore, 2019). Thus, the zebrafish has the theoretical capacity to detect light ranging from UV to IR and across the visual spectrum. Zebrafish cell lines and other teleost cell lines have been used for years in clock studies, yet we do not actually know what opsins are expressed in these cultures.

This raises the question, do zebrafish show such a diversity in opsins in order to be able to capture all photons of any wavelength, such that the system is simply designed to detect the presence or absence of light, regardless of wavelength? Or does this different opsin expression pattern mean that particular organs have specific wavelength sensitivities and therefore differing responses to the environmental light signal?

In this paper, we demonstrate that the light response goes well beyond the visual wavelengths, with both UV and infrared (IR) light pulses having the ability to induce clock gene expression, but interestingly with IR not able to phase shift the molecular clock, at least in cell lines. Furthermore, we show that zebrafish cell lines, rather like the adult tissues (Davies et al., 2015), display a diversity of expressed opsins, a number of which are under clock-control and as such show robust daily rhythms in expression. Whether this transcriptional rhythm in specific opsins translates into matching protein changes is yet to be determined, but it opens up the possibility of a direct temporal regulation of light sensitivity, as well as the more conventional spatial aspects. In this regard, the clock is likely to be gating the process of its own entrainment by regulating expression of components of the light-input pathway; with a specific pathway acting as a zeitnehmer or “time taker” (McWatters et al., 2000).

## Materials and Methods

### Cell Culture

PAC2 and *clockDN* (clock “mutant” cells) cell lines were kept in Leibovitz -15 medium (Gibco) supplemented with 15% FBS (Biowest), 0.05 mg/ml of gentamicin (Gibco) and 1x Penicillin-Streptomycin (Dekens and Whitmore, 2008). Cells were seeded at 50 000 cells/ml and kept at 28°C in a water bath on a 12:12 LD cycle for 3 days before receiving a light-pulsed with different wavelengths (IR 850 nm, red 650 nm, blue 450 nm, UV 350 nm and white 400-700 nm) with an intensity of 200 μW/cm^2^ for 3 h starting light pulse at ZT21 (LED Array Light source, Thorlabs). After a 3-hour light pulse, cells were washed with PBS and homogenised in TRIzol with a cell scraper.

### RNA, cDNA and RT-qPCR

RNA was extracted according to manufacturer’s guidelines (TRIzol, Invitrogen) and the RNA pellet re-suspended in 30 μl of RNase free water (Ambion). 2 μg of RNA was reverse transcribed to cDNA using Superscript II Reverse Transcriptase (Invitrogen), random hexamers (Invitrogen) and oligo dT primers (Invitrogen) according to manufacturer’s protocol. RT-qPCR was performed on a C1000 Touch^TM^ Thermal Cycler with the CFX96^TM^ Optical Reaction Module (Bio-Rad) using KAPA SYBR FAST qPCR mix (Kapa Biosystems) in technical triplicates with gene specific-primers at a concentration of 500 nM. ΔCt was determined using β-actin as a reference gene and relative expression levels were plotted using the ΔΔCt method. Gene specific primers are listed in Table 1.

**TABLE 1:** Gene specific primers used for qPCR.

**Accession no.**	**Current name**	**Alt. Name**	**Forward 5′ -> 3′**	**Reverse 5′ -> 3′**
KT008391	Exorhodopsin		GTA CGC TCC GCT ATC CCA TA	ACG TGT GAA AGC CCC TAC TG
KT008402	Valopa	Val	ACT TCC ACG ACC ACA CCT TC	CGG ATG AGT TTG CAG TAG CA
KT008403	Valopb	Va2	GGC GAG GAT GGT CGT TGT AA	ATG CTG CAT AAG GCG TCC AT
KT008404	parapinopsin-1		CTG TGG TCG TTC ATC TGG AA	GGC CAG ATC TCT GCT GTA CC
KT008405	parapinopsin-2		GCA GCA CTG TAT ACA ACC CCT	ATA CGT CGT CCT CTG AAG GC
KT008406	parietopsin		TGT TGG CGT ATG AGC GTT AT	AGC CAT ACC AAC AGC AGA CC
KT008407	TMTla	tmt6	TGT TAC AGT CGG CTC ATC TGT GCT	ATG TGG TAC TCT CTC CGT CTT GCT
KT008408	TMTlb	tmt9	TGT TGG TGT GTA TGT TCG GGACGA	AGG AGT TGA TGA AGC CGT ACC ACA
KT008409	TMT2a	tmtlO	TTA GTA AGA AGC GGA GCA GAA CCT	ATC CCA TAG GGA TGC AGT GTT GTT
KT008410	TMT2b	tmtl4	CGC AGA GGA GAG AGA ACC AC	TTA GTC CCG TTC TGC CAA AG
KT008411	TMT3a	tmt2	AGG TCG ATG CGA CCA ACT ACA AGA	AAA CAG AGG AGG CAG GGT CCA AAT
KT008412	TMT3b	tmt24	TGC GTG TGG TAC GGT TTC ATC AAT	ATC ATG GTG CAG TAA CGC TCG TAT
KT008413	encephalopsin (opn3)	panopsin	CCCTAT GCT GTG GTC TCC AT	TAG ATG ACG GGG TTG TAG GC
KT008414	neuropsin (opn5)	OPN5ml	ACA CCA TCT GTC GCT CCA TC	CTG CAA ATT GCC CAG TGT C
KT008415	0PN6a	novo3b	GTG GTC AAC ATC CCC TGG AG	ACA ACC AGC CGA GTA TGA GC
KT008416	0PN6b	novo3a	AAT CCA GCC AGG GAG GAG AT	AAG GCG GAC CAC ATG GAA AT
KT008417	0PN7a	novolx	GTT TAA ACA CTA CCCGCG CC	GCTCTG GCTCCA ATT CAG GT
KT008418	0PN7b	novola	TGC TAT ATC GTG CCC TG C TG	CGTACC GTC ACC AGG ATG AG
KT008419	0PN7c	novolb	GTG AAC CTG TCT GTG AGC GA	CTC CCC AAA CAA CCA CCT GT
KT008420	0PN7d	novoly	CTG CCA CTT GGA ATC ATC CT	GCG ACA CAT GCT GCT GTA CT
KT008421	0PN8a	novo2b	TGA CTG ACA TTG GCA TGG CT	TGG TTG AAA GCA GAG GCG AT
KT008422	0PN8b	novo2a	TTC GCT TCA TCG TGT CTT TG	CAG TGG GAA AAT AGC CCA GA
KT008423	0PN8c	novo2x	TGG GCT TTA TCC TTG CCT GG	AGA TGAAGC CTT CTG GTG CC
KT008424	0PN9	OPN5m2	TCA G GG CTT TG T TTT CGG G A	GCA GCG GTC AAG GGA TAT GA
KT008425	Peropsin (RRH)		AGT GGT TGC CAT TGA CCG AT	ATG CGG CCA CAA TCA GAA GA
KT008426	RGR1		CCT GGC TTT CTA CGC CGC AG	GGA CTT GTT CTC AAT AGC AGG ACT CTC
KT008427	RGR2		GAG CAC GTC TAT CAC CAT CAG CT	ACA CCC CAG CCA ATG GCA GG
KT008428	OPN4ml		CGT CAT CAC CTC TGA GTC CA	GCT GGA TTT GTC CCA ACA GT
KT008429	OPN4m2		AGC AAT GCT AGT GGG CAG AA	CGT CTG CTG CAT CCG TTT CA
KT008430	OPN4m3		AAG GCC AAT GGT TCG GAT CC	CCA GGT ATG AGC CTG GAA GA
KT008431	OPN4xl		GCT ACA CCT TGA TGC TCT GC	CTG TTG GAT GAG GGT GGT CT
KT008432	OPN4 × 2		CTT TGT GAA GCA GCA GTC CA	TAT GGA GCC CAG GAC AAA AC
NM_001077297.2	Cry 1a		AGG CTT ACA CAG CAG CAT CA	CTG CAC TGC CTC TGG ACT TT
NM 182857.2	Per2		TGG CTC TGG ACA GAA GTG AG	GGA TGT CTC GAG AAG GCA AC
NM 198143.1	L13		TCT GGA GGA CTG TAA GAG GTA TGC	AG A CGC ACA ATC TTG AG A GCA G
AB042254.1	6–4 photolyase	cry5	TGT GGA TCA TGA GGT TGT CC	TTG ATG GAT GGA CTC GCT TT
NM_001030183.1	Perla	perl	ATC CAG ACC CCA ATA CAA C	GGG AGA CTC TGC TCC TTC T
AF057040.1	Beta a ctin		CGC AAA TAC TCC GTC TGG AT	TCC CTG GAG AAG AGC TAC GA

### Bioluminescent Assays

*Per-1 luciferase* cells, described by Vallone et al. (2004), were plated at 100,000 cell/ml in media (described above) in a white 96-well plate (Greiner) *n* = 16. Cells settled over night at 28°C, and the following day the media was changed for media supplemented with 0.5 nM beetle luciferin (Promega). Plates were sealed with TopSeal clear adhesive from (Perkin Elmer). Bioluminescence was monitored on a TopCount NXT scintillation counter (Packard Instrument Company), in a temperature-controlled chamber (28°C). Cells were entrained on a 12:12 LD cycle and given a light pulse (as described above) at ZT21 after 2 days. Cells were then kept in DD on the TopCount for two more days, at constant temperature, in order measure any phase shift in the gene expression rhythm. Luminescence from the cells was measured in counts per second approximately every hour taking approximately 10 min for a 96-well plate to be read.

### Statistical Analysis

*T*-tests, ANOVAs and post-test were performed with the standard add-in software in Excel. Alpha was set at 0.5. Tukey numbers were calculated using values from a standard Tukey table.

## Results

### Opsin Expression in Cell Culture

**FIGURE 1:**
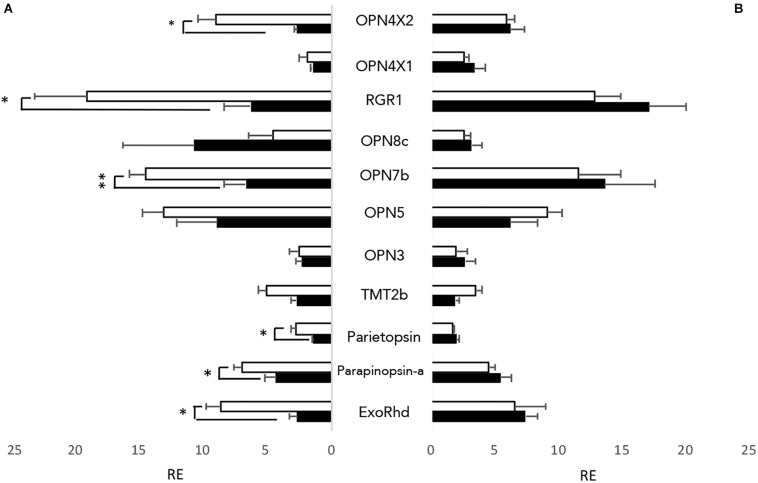
Non-visual opsins expressed in zebrafish cell lines. All 32 non-visual opsins were explored by RT-qPCR in entrained zebrafish cell lines at opposite times of day; ZT3 (white) and ZT15 (black). 11 opsins showed detectable expression levels, using a cut-off value of <C_q_ 30. **(A)** Opsins expressed in PAC2 cell lines. **(B)** Opsins expressed in *clockDN* cell lines. Opsin expression is plotted relative to the lowest detectable opsin, with error bars depicting SEM. An unpaired students *t*-test was used to assess if the opsins expressed show a time of day specific expression pattern. ^∗^*p* < 0.05 and ^∗∗^*p* < 0.001 (*n* = 4).

As well as having directly light sensitive organs, zebrafish cell lines, typically generated from early stage larvae such as the PAC2 cell line, are also directly light responsive (Whitmore et al., 2000). To examine which opsins are present in the cells, both PAC2 cells, and transformed cells expressing a *clock*-dominant negative construct (*clock*DN cells) were kept on a 12:12 light dark cycle at constant temperature, and cells were harvested at ZT3 and ZT15. Both cell lines express opsins from all classes of non-visual opsins, with a total of 11 out of 32 non-visual opsins expressed at a detectable level (C_t_ lower than 30) (Figure 1). There is no apparent difference between PAC2 and the *clockDN* lines in opsin expression pattern. By comparing the expression pattern at two different times of day, we also observed that half of the opsins show a day-night difference in expression pattern in PAC2, but not *clockDN* cells, which shows that some opsin expression is clock controlled (Figure 1). Interestingly, two forms of OPN4 are expressed in these zebrafish cell lines and one, OPN4 × 2, shows a strong day-night difference in expression. This is also the case for exo-rhodopsin, which is typically considered to be a pineal specific photopigment. RGR1, a putative photoisomerase, also shows robust daily changes, and is the most abundant transcript.

### Impact of Light on Clock Genes in Cells

**FIGURE 2:**
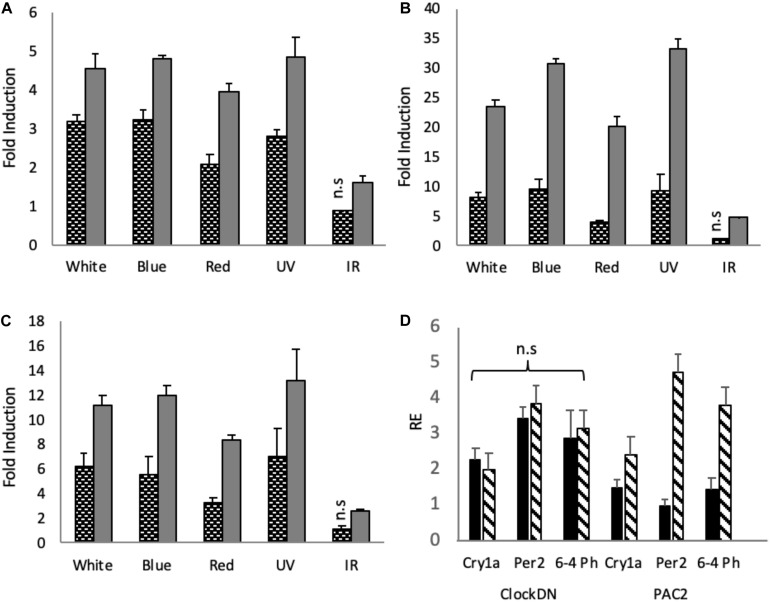
Light induction by monochromatic light-pulses in PAC2 and *clockDN* cell lines. Zebrafish cell lines were maintained on a 12:12 light-dark cycle before being given a 3-hour light pulse of varying wavelengths. *B*lack and white bars represent *clockDN* cell line expression, whilst solid grey represent PAC2 cell line expression. **(A)** Light induction of *cry1a*. **(B)** Light induction of *per2*. **(C)** Light induction of 6–4 Photolyase. **(A–C)** is plotted as fold induction relative to dark control. **(D)** Dark controls (black bars) vs. IR monochromatic light pulse (striped bars) plotted relative to lowest expressed gene (PAC2 *per2* DD) in *clockDN* and PAC2 cells. Significance was addressed with an one-way ANOVA (α = 0.05) for each light-pulse, cell line and gene, followed by a Bonferroni post-test. All light pulses give a significant increase of *p* < 0.05 unless marked on the graph (*n* = 3).

To explore how monochromatic light of selected wavelengths impacts gene expression in zebrafish cell lines, *ClockDN* and *PAC2* cells were entrained, like the organs, on a 12:12 LD cycle at constant temperature and given a monochromatic light pulse for 3-hour at ZT21, when cells are most light responsive (Tamai et al., 2005). Using RT-qPCR, we examined the effect of these light pulses on different, well-established light responsive clock genes, such as *cryptochrome1a* (*cry1a*) and *period2* (*per2*), as well as the light induced DNA repair gene, *6-4 Photolyase* (*6-4 Ph*) (Tamai et al., 2007; Vatine et al., 2009). In *PAC2* cells, white, blue and UV light pulses of the same intensity give very similar induction in all genes explored, whilst red generates a slightly smaller, yet not statistically different induction (Figures 2A–C). IR pulses give the smallest induction of the genes explored. In *cry1a* we see a significant 1.6-fold induction, as opposed to ∼4-fold induction by the other wave lengths (Figure 2A). For *per2* IR give a ∼5-fold induction as opposed to 20–30-fold by the other wavelengths (Figure 2B). Finally, IR gives a 2.6-fold induction as opposed to up to 13-fold induction, by the other wavelengths (Figure 2C). IR does indeed induce significant induction of the light sensitive clock and a DNA repair gene. However, compared to the other wavelengths, it is between 2.5 and 6 times less potent, depending on the gene in question.

*ClockDN* cells show a reduced fold induction to all the wavelengths (Figures 2A–C). The raw C_t_ values seen in *clockDN* and PAC2 cells are, however, very similar when given a light-pulse. These clock mutant cells show a higher basal DD expression of the clock and DNA repair genes, and thus the fold induction is subsequently lower (Supplementary Figure S1). This is particularly evident when giving an IR light pulse (Figure 2D).

### Phase Shift in Cell Culture

**FIGURE 3:**
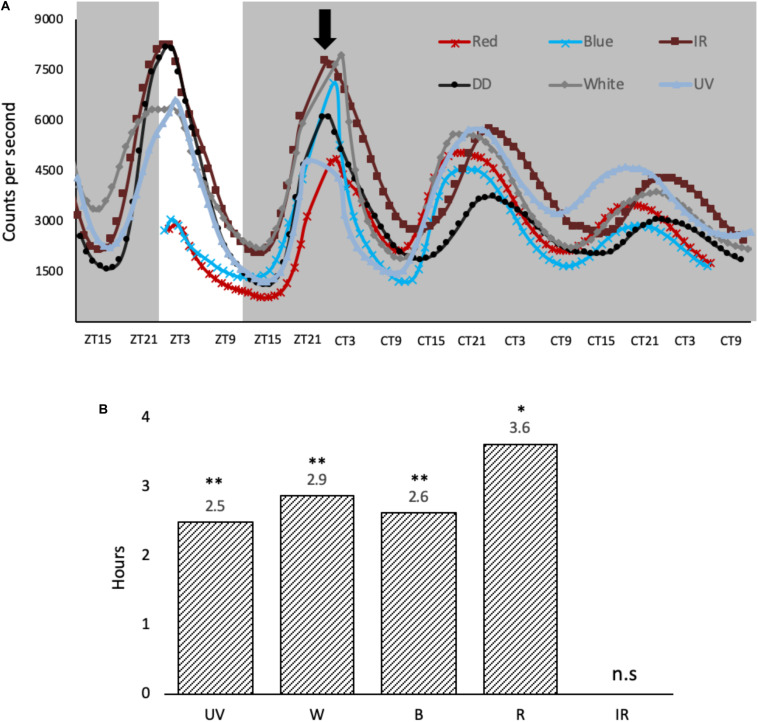
Light-induced phase shifts in response to monochromatic light-pulses in *per1-luciferase* zebrafish cells. **(A)** Cells were entrained for 3 days on a 12:12 LD cycle, before they were given a variety of monochromatic light pulses at ZT21, denoted by black arrow. The cells were kept in constant dark over two subsequent days. **(B)** Light pulses cause a phase advance in hours, determined at the half maximum between peak and trough vs. no LP controls. A two-way ANOVA (time, wavelength) was used to determine significant variation between samples. Amplitude and baseline were detrended using BioDare2 (Zielinski et al., 2014), and a Tukey post-test was used to determine significance in phase shift relative to DD control. ^∗^*p* < 0.01 and ^∗∗^*p* < 0.001 (*n* = 8).

To explore how the monochromatic light phase shifts the molecular clock in cell culture, *per1-luciferase* luminescent reporter cells (Vallone et al., 2004) were entrained for 3 days at 28°C and pulsed the same way as the cells described above (Figure 3A). *Per1* luminescent traces were then monitored for 2 days post light pulse in DD using the Packard TopCount. UV, blue, red and white light are all capable of causing a phase advance in the cell culture clocks when light is applied at this particular time in the cycle (ZT21) (Figure 3B). Interestingly, a 3-hour light exposure of IR light does not give a phase shift regardless of the acute molecular response to this light signal, increasing both *cry1a* and *per2* expression, a result which is worthy of further discussion.

## Discussion

### Cells Show a Diversity in Opsin Expression Patterns

Zebrafish cell cultures have long been used because of their direct light sensitivity. However, the opsin composition of these cells lines has never previously been published. We, therefore, performed RT-qPCR on PAC2 cell lines to explore what opsins are expressed. With RT-qPCR and setting a cut off value at C_q_ 30 as a measure of “no expression,” we can identify the presence of 11 out of 32 non-visual opsins (Figure 1A). Interestingly, 6 of these opsins show a clear day-night difference in expression and appear to oscillate. All of these opsins show higher levels of expression during the day time-point compared to night. To explore this further, we therefore also examined expression in the *ClockDN* cell line, lacking a functional circadian clock, to manifest whether this difference is light-driven or clock-dependent. Interestingly, the *ClockDN* cells express the same specific opsins exactly, but they no longer oscillate (Figure 1B), which supports the idea that expression of these opsins is directly clock controlled and not directly light-driven. Furthermore, averaging expression of ZT3 and ZT15, there is no significant difference in the amount of transcript produced in the two different cell lines. The opsin expression profile in cells does not resemble any particular tissue type that we know of today. However, it is worth noting that the cell line express one opsin from all the opsin families, like most tissue types, and thus possess G_q_, G_t_, G_i_, and G_o_ coupled opsins, as well as a putative photoisomerase (RGR1). The cell line should, therefore, be able to signal through the same pathways in response to light as any other fish tissue.

### Monochromatic Light (350–650 nm) Are Potent Inducers of Light Responsive Genes in Cell Culture

Expression of the light responsive clock genes in cell culture is rather flat with a broad response to white, blue and UV light. There is a slight but statistically significant drop in the response to red light in the cells, and a marked drop in the response to IR (Figure 2).

6*–*4 photolyase catalyses the photo-reversal of the (6–4) dipyrimidine photoproducts induced in DNA by ultraviolet light (Zhao et al., 1997). A simple prediction might be that UV/blue light should be more efficient at inducing expression of this DNA repair enzyme. However, this does not appear to be the case, with red light and even IR light able to increase transcript levels. Red light photons have lower energy than blue light photons, therefore, the same intensity of blue and red light will have different number of emitted photons. Consequently, one hypothesis is that the opsins simply “count” photons, not the energy of the photons they absorb, meaning that the zebrafish cell simply wants to know whether there is light present or not. To address such issues, these experiments will need to be repeated considering aspects of photon flux over a wider range of light intensities. Of course, it may be biologically essential to activate expression of your DNA repair machinery in the presence of light, regardless of the subtleties of the specific wavelength, and of course the 6–4 photolyase protein itself absorbs light to perform its role in replacing cross-linked nucleotides. It is this aspect of light driven DNA repair that is most likely to be wavelength sensitive.

Comparing cells without a functional clock to “wild type” cells, we also see that the fold induction of genes in response to light is lower, due to a higher basal transcription of these target genes in DD. *ClockDN* cells show a higher basal DD expression of the clock and DNA repair genes, and the fold induction is subsequently lower (Figures 2A–C). This is interesting, as it demonstrates the steady state expression levels that these genes reach in a non-rhythmic mutant background. The absolute expression remains the same (Supplementary Figure S1). Interestingly, the high basal level of transcript means that there is no induction of light responsive clock genes in the *clockDN* cells in response to IR.

### UV- Red Light Can Alter Gene Expression and Phase-Shift Cell Lines

The impact of “visible” wavelengths of light (380–740 nm) on the zebrafish clock system has been described in numerous previous studies. However, exploring this phenomenon outside of the visual spectrum are rarely performed in fish. UV light of 350 nm (UVA) has a clear impact on gene expression and can clearly phase shift the circadian clock in cell lines. Perhaps this is not so surprising from what we now know about zebrafish photobiology. After all, 350 nm is only 50 nm below the violet/blue wavelengths that can so robustly impact the clock in an aquatic organism. In future, it would be interesting to try wavelengths at the more extreme end of the UVA range and well away from the visual spectrum. This UV response also fits well with the previously determined absorption spectra for purified opsin proteins, which reveals a wide sensitivity in the UV/blue wavelengths (Davies et al., 2015).

The impact of these monochromatic light pulses was explored using our luminescent reporter cell lines. At the phase (ZT21) and intensity used, each wavelength generated a very similar phase advance in the rhythm, including UV light pulses, but not IR at 850 nm (Figure 3A). This similarity in size of phase advance correlates well with the similarity in molecular response, induction of *cry1a* and *per2*, seen in the cell lines (Figure 2). Furthermore, using a Tukey post-test, there is statistical difference in the size of phase shift generated by each of these light pulses (except between blue and UV) (Figure 3B). Since this difference in shift is so small, it may be due to the sampling frequency (plate counted once an hour) rather than real difference, thus we do not speculate any further.

Compared to previous studies on phase shifting in zebrafish cell lines, in response to white light, the size of the phase shift is relatively small and is actually a phase advance rather than a large phase delay previously reported (Tamai et al., 2007). The reasons for this simply relate to the differences in light intensity used. Early studies applied light at 5000 μW/cm^2^, compared to the 200 μW/cm_2_ used in this study. Consequently, the Type 0 PRC previously reported switches to a more “standard” Type 1 PRC as the lower light intensity, as historically seen in many previous studies. Interestingly the switch in PRC amplitude, therefore, occurs between these two intensities, and strongly suggests that fish under natural conditions, as a diurnal animal, will be “working with” a Type 0 PRC.

The response to infrared light was not expected. As a stimulus, it is generally avoided in clock studies, due to the strong link with temperature effects/artefacts and the ability of temperature pulses to phase shift the circadian clock. It is a stimulus typically one aims to control against in circadian analysis. Yet the response to IR when controlling for temperature, of the zebrafish clock system is very interesting. IR of 850 nm can clearly cause specific transcriptional changes in zebrafish cells. Obviously, in our experiments we aimed to avoid the thermal heating effect of IR exposure, and none was detected in our cultures. Equally, the IR light pulse did not phase shift the clock, which has been shown to be robustly phase-shifted by temperature pulses (Lahiri et al., 2005). For cells in culture, IR causes a small, yet significant induction of *cry1a* and *per2*, however, there is no subsequent phase shift. As mentioned earlier, IR is up to 6 times less potent in cell lines, and the reduced induction of *cry1a* and *per2* compared the other wavelengths may not be sufficient to cause a downstream phase shift in these studies. Of course, it could also be the fact that *cry1a* and *per2* are not as central to phase shifting the teleost clock as has been previously proposed.

How IR signals to cells in a meaningful way is a fascinating question. It could be through mitochondrial-driven processes or it could be through the “re-purposing” of the mass of opsin in fish to perform other key sensory roles. If fish opsins are acting as “thermal” or IR sensors, as has been proposed in Drosophila, then this opens up a whole new world of interesting (fish) biology.

In this study, we have shown that the spectral sensitivity of zebrafish cell lines extends beyond the classically perceived “visual” wavelengths of light and that supporting this wide spectral sensitivity, these cells express a large number of opsins. Furthermore, the clock itself regulates the temporal expression of these opsins, raising the interesting possibility that the clock itself controls light input to the pacemaker – the zeitnehmer concept that has so eloquently been described for plant clock systems.

## Data Availability Statement

The raw data supporting the conclusions of this article will be made available by the authors, without undue reservation.

## Author Contributions

Both authors performed the experiments, analysed the data, wrote the manuscript and contributed to the article and approved the submitted version.

## Conflict of Interest

The authors declare that the research was conducted in the absence of any commercial or financial relationships that could be construed as a potential conflict of interest.

## Supplementary Material

FIGURE S1 Monochromatic light pulses in PAC2 vs. *clockDN* cell lines. *ClockDN* cell lines (light grey) show increased basal expression of all genes explored in the dark control compared to PAC2 (dark grey). Significance was addressed with a one-way ANOVA (α = 0.05) for each light-pulse, cell line and gene, followed by a Bonferroni post-test. All light pulses give a significant increase of *p* < 0.05, except *clockDN* DD vs. IR (*n* = 3).
