# Supplementary figures and images for: Zebrafish Circadian Clock Entrainment and the Importance of Broad Spectral Light Sensitivity
Source: Front Physiol. 2020 Aug 14;11:1002. doi: 10.3389/fphys.2020.01002 (PMC7456917; doi:10.3389/fphys.2020.01002)

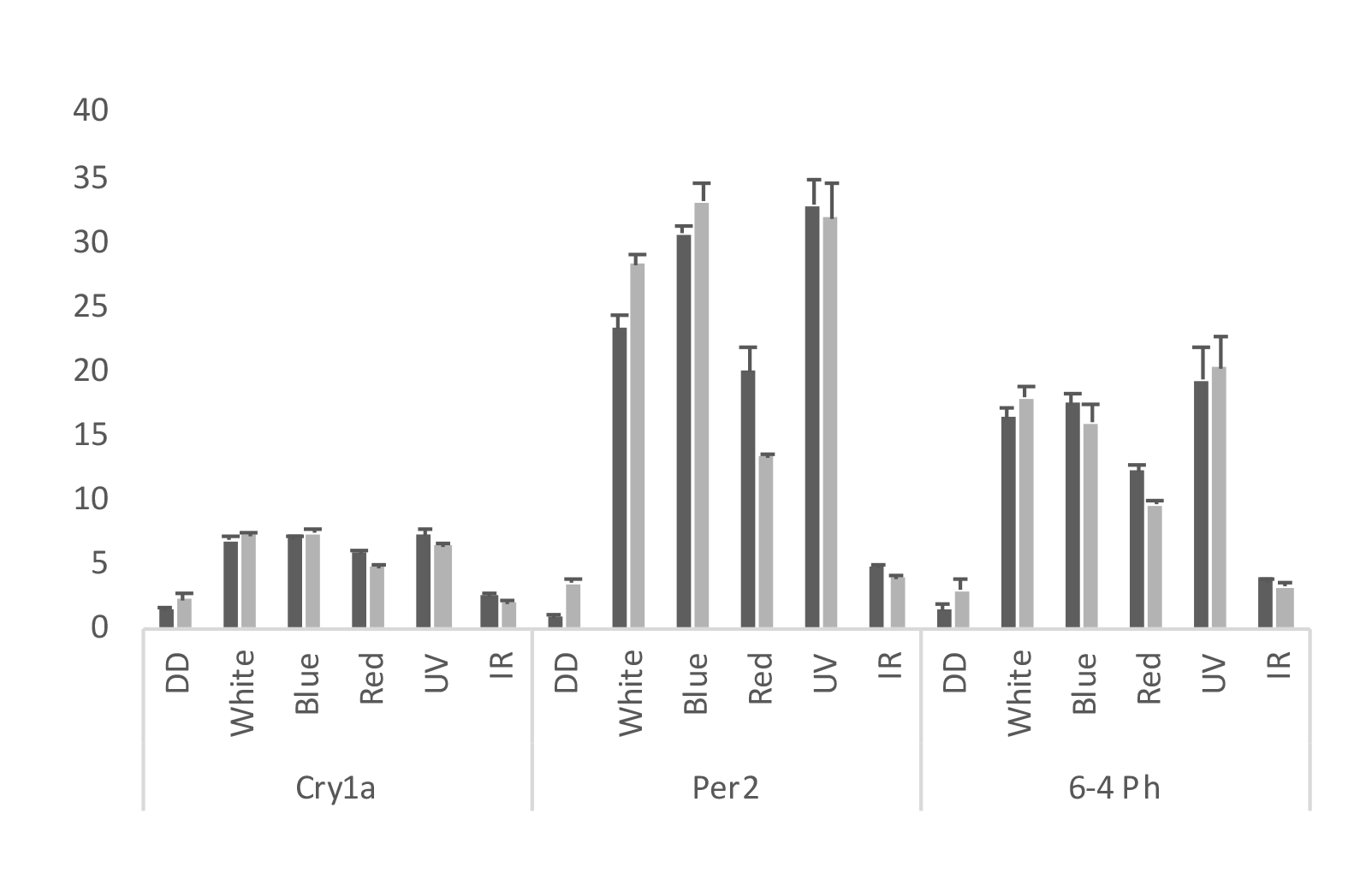

Supplement: FIGURE S1 — Monochromatic light pulses in PAC2 vs. clockDN cell lines. ClockDN cell lines (light grey) show increased basal expression of all genes explored in the dark control compared to PAC2 (dark grey). Significance was addressed with a one-way ANOVA (α = 0.05) for each light-pulse, cell line and gene, followed by a Bonferroni post-test. All light pulses give a significant increase of p < 0.05, except clockDN DD vs. IR (n = 3). [file Image_1.tif]
